# Clinical assessments and care interventions to promote oral hydration amongst older patients: a narrative systematic review

**DOI:** 10.1186/s12912-016-0195-x

**Published:** 2017-01-17

**Authors:** Lloyd L. Oates, Christopher I. Price

**Affiliations:** 1 Northumbria Healthcare NHS Foundation Trust, Stroke Research, Wansbeck General Hospital, Woodhorn Lane, Ashington, Northumberland NE63 9JJ UK; 2 Newcastle University Institute for Ageing, Newcastle University Stroke Research Group, 3-4 Claremont Terrace, Newcastle upon Tyne, NE1 7RU UK

**Keywords:** Dehydration, Drinking, Fluid therapy, Nursing care, Risk assessment

## Abstract

**Background:**

Older patients in hospital may be unable to maintain hydration by drinking, leading to intravenous fluid replacement, complications and a longer length of stay. We undertook a systematic review to describe clinical assessment tools which identify patients at risk of insufficient oral fluid intake and the impact of simple interventions to promote drinking, in hospital and care home settings.

**Method:**

MEDLINE, CINAHL, and EMBASE databases and two internet search engines (Google and Google Scholar) were examined. Articles were included when the main focus was use of a hydration/dehydration risk assessment in an adult population with/without a care intervention to promote oral hydration in hospitals or care homes. Reviews which used findings to develop new assessments were also included. Single case reports, laboratory results only, single technology assessments or non-oral fluid replacement in patients who were already dehydrated were excluded. Interventions where nutritional intake was the primary focus with a hydration component were also excluded. Identified articles were screened for relevance and quality before a narrative synthesis. No statistical analysis was planned.

**Results:**

From 3973 citations, 23 articles were included. Rather than prevention of poor oral intake, most focused upon identification of patients already in negative fluid balance using information from the history, patient inspection and urinalysis. Nine formal hydration assessments were identified, five of which had an accompanying intervention/ care protocol, and there were no RCT or large observational studies. Interventions to provide extra opportunities to drink such as prompts, preference elicitation and routine beverage carts appeared to support hydration maintenance, further research is required. Despite a lack of knowledge of fluid requirements and dehydration risk factors amongst staff, there was no strong evidence that increasing awareness alone would be beneficial for patients.

**Conclusion:**

Despite descriptions of features associated with dehydration, there is insufficient evidence to recommend a specific clinical assessment which could identify older persons at risk of poor oral fluid intake; however there is evidence to support simple care interventions which promote drinking particularly for individuals with cognitive impairment.

**Trial registration:**

PROSPERO 2014:CRD42014015178.

**Electronic supplementary material:**

The online version of this article (doi:10.1186/s12912-016-0195-x) contains supplementary material, which is available to authorized users.

## Background

Older adults are susceptible to dehydration due to acute and chronic health problems, which impair thirst, reduce the ability to drink sufficiently and/or increase urinary, skin and respiratory fluid loss [1]. During hospitalisation negative fluid balance often accompanies infection and is independently associated with poorer outcomes [2–5], longer length of stay and greater costs [6–8]. In England the National Institute for Healthcare and Care Excellence has estimated that the annual impact from acute kidney injury is up to £620 million [7] and that 12,000 cases could be avoided by more pro-active fluid management amongst vulnerable groups such as older adults. Specific associations with dehydration have already been described with acute stroke [9], and admission from a long term care setting [10]. Although it is a clinical priority to recognise and address risks of insufficient oral fluid intake, there is no standardised nurse-led assessment or formal bedside response protocol commonly applied. A recent Cochrane review [11], of studies to identify impending and current water loss in an older people recommended that for clinical practice “there is no clear evidence for the use of any single clinical symptom, sign or test of water-loss dehydration in older people. Where healthcare professionals currently rely on single tests in their assessment of dehydration in this population this practice should cease because it is likely to miss cases of dehydration (as well as misclassify those without water-loss dehydration).” Previous studies have recommended combining various data items to identify individuals, who may need fluid support interventions. Some studies have often confused a risk of inadequate fluid intake with characteristics already indicating a dehydrated state or relied upon serial laboratory measures of renal function and osmolality [2, 12]. In the absence of a single test/symptom based upon an objective reference standard of hydration status, our aim was to look qualitatively at the evidence for any assessment (including multiple combinations of factors) and matching intervention which could be easily used at the bedside specifically to reduce the risk of dehydration (not to identify an already dehydrated state). This would not be restricted to studies attempting to validate against laboratory measures of fluid status. In order to make recommendations regarding care processes during hospitalisation, studies would be selected from institutional settings, including care homes.

## Methods

Using PRISMA guidelines [13] articles published in English were sought where the main focus was use of a hydration/dehydration assessment in an adult population with/without a care intervention to promote oral hydration. Review articles were included where a new assessment tool was developed as a result of findings. Articles were excluded which described single case reports, laboratory results only, technology which was not integrated into a clinical score e.g. bioelectrical impedance analysis (BIA) or non-oral fluid replacement in patients who were already dehydrated. Interventional studies were included if the intention was specifically to promote oral hydration rather than nutritional intake in general.

A search of electronic databases (MEDLINE, EMBASE and CINAHL) was conducted using keywords: dehydration, prevention, assessment, screening, hospitals and care homes. The reference lists of identified papers were cross-referenced for new articles. Grey literature (non published academic work, hospital protocols and existing dehydration assessment tools) was sought through Google and Google Scholar. Interventional studies were included if the intention was specifically to promote oral hydration rather than nutritional intake in general. A structured data extraction and quality appraisal form was used for information extraction including: design, population and identification, method of data collection, results, ethical considerations, key ideas and author’s conclusions [14–16]. The first author (LO) screened initial titles and abstracts. Two authors (LO,CP) independently reviewed full text articles. Differences were resolved in scheduled meetings. Due to the mixed nature of the studies and uncertainties about the generalizability of different settings, results are presented as a narrative synthesis and no additional analysis was performed. The protocol was registered with the PROSPERO International prospective register of systematic reviews (PROSPERO 2014:CRD42014015178). Fuller details of the search methods are available from the corresponding author.

## Results

### Search results

**Fig. 1:**
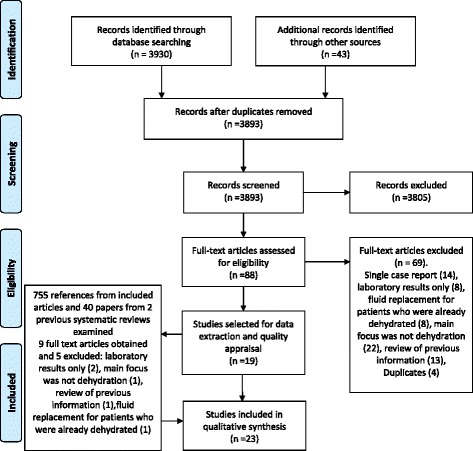
Search results flow diagram. The figure shows the flow diagram of the search results under PRISMA headings of identification, screening, eligibility and included

Figure 1 describes the study selection process. A total of 3973 articles were identified, after removing duplicates 3893 remained. Out of 3893 retrieved articles, 3805 were excluded by title and/or abstract, 69/88 full text articles were excluded because they were duplicate or single case reports, did not focus on dehydration prevention or oral fluid risk management and/or only considered additional non-oral fluid replacement strategies for patients who were already known to be dehydrated. Within the reference lists of the remaining articles a further four relevant papers were identified.

Table 1 describes a summary of the extracted data. Of the 23 articles there were eight intervention studies, six non-systematic literature reviews, two guidelines, one assessment proposal, two audits, one multi-phase project summary and three surveys. Publication dates ranged from 1984 to 2016. Countries of origin were USA (nine), UK (eight), Australia (five) and Italy (one). Comparison of quality was challenging due to the variable nature of the articles; however most had a clear stated aim and identified their target setting. The search did not identify adequately powered randomised controlled trials and large prospective observational studies. The individual risk factors for poor hydration reported across the 23 included articles are summarised below. To describe the clinical context of each assessment or intervention, each article has then been placed into one of five groups: identification checklist/chart (five), identification checklist/chart with care intervention (five), identification by urinary inspection (two), promotion of oral intake (four), professional knowledge/awareness improvement (seven), as seen in Table 1.

**Table 1:** Summary of extracted data

Group	First author, Year, Country	Clear statement of aims	Article Type	Setting	Purpose	Participants	Mean Age	Female (%)	Results/Recommendations			
Identification checklist/chart	Vivanti (2010) Australia [17]	Y	Observational prospective analysis	Hospital	Screening questions and clinical parameters in hydrated and dehydrated patients.	86 (36 dehydrated)	78.6	54.7	Inter rater repeatability 70–95% agreement.			
										Sensitivity	Specificity	
									Tongue dryness	64%,(95% CI 54–74%)	62%, (95% CI 52–72%)	
									Pain interference	83%, (95% CI 76–90%)	32%, (95% CI 23–43%)	
									Drop in systolic BP	69%, (95% CI 59–79%)	56%, (95% CI 46–66%)	
									Skin turgor	44% (95% CI 34–54%)	65% (95% CI 55–75%)	
									The authors identified tongue dryness as a clinical feature to identify dehydration amongst older people. Further studies were recommended.			
Identification checklist/chart	Vivanti (2008) Australia [18]	Y	Observational prospective analysis	Hospital	Over 40 clinical parameters were explored in hydrated and dehydrated patients.	43 patients 8 Focus Group 9 Interviews	78.3	65	Presence of mild dehydration:			
									Tongue dryness	71.4%,(OR 4.4 (95% CI 0.8–26.1))		
									Tongue furrow	57.1%, (OR 3.0 (95% CI 0.5–15.8))		
									Dry oral mucous membrane	57.1%, (OR 2.3 (95% CI 0.4–12.0))		
									Tissue turgor hand	88.7%, (OR 2.6 (95% CI 0.2–24.6))		
									Tissue turgor sternum	14.3%, (OR 5.8 (95% CI 0.3–106.4))		
										Dehydrated	Hydrated	p value
									Systolic BP standing drop	20.1 ± 20.8 mmHg	2.1 ± 19.0 mmHg	0.03
									BMI	20.0 ± 3.0	27.5 ± 6.2	0.03
									Weight	46.7 kg	71.5 kg	0.04
									The authors reported that physical, rather than biochemical, parameters more often identified mild dehydration.			
Identification checklist/chart	Bulgarelli (2015) Italy [34]	Y	Observational prospective analysis	Hospital	Evaluation of the DRAC	21 (received checklist)	80	54.7	Patient’s scores evaluated within 3 days of admission and at discharge. Scores on the DRAC did not significantly change between admission and discharge and were not correlated with laboratory measures of dehydration.			
Identification checklist/chart	Mentes (2011) USA [26]	Y	Observational retrospective analysis	Nursing Home	Evaluated the DRAC using a factor analysis, and multiple logistic regression.	133 (9 Nursing Homes)	83.1	56.4	40 items were reduced to 17 based on frequency distribution. The remaining factors were examined for their association with dehydration, which varied from −0.012 (female gender) to 0.567 (urinary incontinence). See Table 3 for the factors included. Overall there was low to moderate association with dehydration. An increased number of risk items indicate a greater overall risk.			
Identification checklist/chart	Wotton (2008) Australia [19]	Y	Review	N/A	Reviewed risk factors and explored the reliability of clinical signs.	N/A	N/A	N/A	The authors concluded that the management of fluid and electrolyte balance requires a complex mixture of skills including knowledge, expertise and an understanding of the underlying physiological principles of fluid balance in the body. The use of multiple patient assessment cues should be used by nurses to differentiate between and respond to the various causes of dehydration. Actions include education for older adults on adequate fluid intake, visual reminders to drink, increased offering of fluids between meals and special drinking apparatus or swallowing exercise.			
Identification checklist/chart with care intervention	Food First team (2012) United Kingdom (England) [20]	N	Clinical guideline	Hospital	Reporting of a checklist with an accompanying response protocol.	N/A	N/A	N/A	Recommendations were to assess 24 h fluid intake, urine colour, and symptoms associated with dehydration risk before formalising an individual hydration plan.			
Identification checklist/chart with care intervention	Keller (2006) Australia [32]	Y	Audit	Nursing Home	Use of an audit tool to measure current practice against best practice.	Pre-audit 96 Post-audit 15	65<	Not reported	96 records were audited showing an increase from 40% to 100% in risk identification, but there was no improvement in hydration management. The audit tool was useful in identifying current practice, to facilitate change with the aim to improve clinical outcomes in residential homes.			
Identification checklist/chart with care intervention	Zembrzuski (1997) USA [21]	Y	Review	N/A	Reporting of a broad approach to hydration management.	N/A	N/A	N/A	Approach categories included: administration, work with clinical staff and in service education activities. An assessment tool, administrative and education guidelines and brain storming sheets are included to allow the reader to initiate a prevention of dehydration programme. Items on the assessment chart had equal weighting. A higher risk of dehydration was assumed if more factors were present.			
Identification checklist/chart with care intervention	NHS East of England (2011) United Kingdom (England) [22]	Y	Clinical guideline	N/A	Development of an information package including policy guidance for: assessing, planning, delivering, evaluating and recording fluid care.	N/A	N/A	N/A	Audit results indicated inadequate staff knowledge about the assessment and management of fluid volumes. Nine principles were developed to improve hydration management. The bundle included urine colour charts and a drinks tick chart for patients. Staff were provided with a range of tools for auditing and recording hydration.			
Identification checklist/chart with care intervention	Mentes (2000) USA [23]	Y	Review	N/A	Development of a protocol for healthcare staff to identify dehydration and provide strategies to promote hydration.	N/A	N/A	N/A	Hydration management should be defined in three stages: risk identification, intervention and review. Individual management plans should include a statement regarding the frequency that patients are to be offered drinks. The DRAC was divided into the following sections: Significant health conditions/situations, intake behaviours, medications and laboratory results with female gender and aged over 85 also higher risk factors.			
Identification by urinary inspection	Rowat (2011) United Kingdom (Scotland) [35]	Y	Observational prospective analysis	Hospital	Evaluation of urine colour and urine specific gravity as an early indicator of dehydration when compared to blood indicators in stroke patients.	20	79	55	Dehydrated patients had a non-significant higher median test strip Usg and refractometer Usg and Ucol than those hydrated. The within-subject agreement between the refractometer Usg and nurse’s opinion was 84%. Refractometer agreed with 40% of urine test strip Usg. Agreement between refractometer Usg and Ucol was 67%. The results do not support the use of the urine test strip urine specific gravity as an early indicator of dehydration.			
Identification by urinary inspection	Mentes (2006) USA [33]	Y	Descriptive correlation	Nursing Home	Evaluation of urine colour as a valid indicator of dehydration.	98 (7 nursing homes)	84	53	Urine colour averaged over several readings provides another tool in assessing dehydration status with individuals with adequate renal function when compared against urine specific gravity. The authors concluded that the method is low intensity and low cost but further study is needed to improve generalizability.			
Promotion of oral intake	Wakeling (2011) United Kingdom [27]	Y	Audit	Hospital	Evaluated whether using a drinking aid (sports bottle/bite valve straw) that attached to the patients bed could improve fluid monitoring.	313 patients 23 Staff	16–98	Not reported		Length of stay (days)	Dehydration	Infections
									Pre-intervention	41	31	28
									Post intervention	33	1	0
									Patient questionnaires −142 distributed, 44 returned suggesting the drinking aid was easy to use.			
Promotion of oral intake	Robinson (2002) USA [29]	Y	Quasi Single Subject ABA	Nursing Home	Evaluation of a hydration assistant, individualised care plan and a colourful beverage cart with a selection of drinks.	51	83.5	84.3	Post-intervention significant improvements seen in TBW (*p* = 0.001), bowel movements (*p* = 0.04), reduction in laxative use (0.05), decline in number of falls (0.05) and decline in costs (0.05). 53% of participants always consumed an additional 8 oz of beverage mid-morning and mid-afternoon. The authors conclude that providing two additional glassed of fluid per day is a simple intervention and can make an important difference in a resident’s quality of life.			
Promotion of oral intake	Simmons (2001) USA [30]	Y	Controlled clinical intervention trial	Nursing Home	Evaluation of verbal prompts and patient’s drink preference.	63 (2 nursing homes)	IG 88.9 CG 86.3	IG 92 CG 67	Significant correlations with fluid intake changes were: age (*r* = −.32, *P* = .015), BMI (*r* = .45, *P* = .001), and MMSE (*r* = − .494, *P* = .000). The intervention was effective in increasing fluid intake. Verbal prompting alone was effective in improving fluid intake in the more cognitively impaired residents, whereas preference compliance was needed to increase fluid intake among less cognitively impaired residents.			
Promotion of oral intake	Spangler (1984) USA [36]	N	Intervention RCT crossover	Nursing Home	Evaluation of a cart equipped with liquids and toileting equipment, aimed to decrease incontinence and improve hydration.	30 Interviewed 16 Selected	59–96	87.5		Pre intervention	Post intervention	
									Dehydrated	25%	0% (two participants over hydrated)	
									There was an overall decrease in dehydration, and significant improvements in mean urinometer scores (p < 0.002)			
Professional knowledge/awareness improvement	NHS Kidney Care (2012) United Kingdom [37]	Y	Survey	Hospital	Survey of use and impact of a poster campaign.	53 Trusts	N/A	N/A	Survey had a 33% response rate. 79.2% had received the poster pack and 69.8% had displayed them. Posters were displayed on wards 54.7% of the time. 45.3% of respondents had a policy to monitor hydration, 15.1% had a policy but felt it needed updating, 28.3% did not respond to the question and 11.3% of respondents did not have a policy to monitor hydration. Challenges preventing hydration monitoring− 22.6% compliance of documentation. 17% keeping up to date with current practice and 11.3% raising staff awareness on the importance of monitoring. 64.2% felt staff would benefit from more training.			
Professional knowledge/awareness improvement	McIntyre (2012) United Kingdom [31]	Y	Audit/Survey	Hospital	Implementation of the NHS East of England Intelligent fluid management bundle.	0	N/A	N/A	8 of 11 NHS trusts were using the tool. The five key points introduced included: All patients should have their fluid needs assessed, a plan should be made for each patient, fluid intake should be managed continuously, hydration should be reviewed for early detection of deterioration, and education for all should underpin the principles of successful fluid management.			
Professional knowledge/awareness improvement	NPSA and RCN (2007) United Kingdom [38]	Y	Clinical guideline	Hospital	Development of a toolkit to improve best practice amongst hospitals in the UK.	N/A	N/A	N/A	The toolkit comprised factsheets; checklists and advice presented in 11 sections from the RCN nutrition now campaign.			
Professional knowledge/awareness improvement	Kositzke (1990) USA [24]	Y	Review and Case Study	Hospital	Identification of risk factors and assessment of hydration intervention.	1	89	0	In the case study presented, success was signalled by normal skin and tongue turgor, urine output in adequate amounts with normal specific gravity, pulse, blood pressure and absence of risk factors.			
Professional knowledge/awareness improvement	Beattie (2013) Australia [39]	Y	Survey	Nursing Home	Survey of staff knowledge about nutritional needs, mealtime practices and attitudes towards mealtime practices.	76	Not reported	90	38% of staff reported conducting fluid intake/output assessments. Nursing staff scored higher.			
										Knowledge (% correct)		
									Overall	47		
									Malnutrition risk factors	76		
									Hydration status	63		
									Fluid requirements	15		
									The results demonstrated a need to enhance awareness and assessment skills.			
Professional knowledge/awareness improvement	RCN Nutrition Now Campaign (2007) United Kingdom [25]	Y	Summary	N/A	Summary of the NPSA and RCN Nutrition now campaign.	N/A	N/A	N/A	Recommended 20 points to encourage water consumption. Some of these included: using visual aids for patients at increased risk, to offer larger volumes of fluid when giving out medication and to include patients, family and friends in hydration promotion.			
Professional knowledge/awareness improvement	Mentes (2013) USA [28]	Y	Review	N/A	Summary of an evidence based practice guideline “Hydration Management Protocol”	N/A	N/A	N/A	The article presents a summary of previously published information to increase the readers knowledge of definitions, risk factors and intervention included in the DRAC.			

*DRAC* dehydration risk appraisal checklist, *RCN* Royal College of Nursing, *NPSA* National patient safety agency

### Individual risk factors

The most common clinical factors associated with dehydration reported by the different literature sources are listed in Table 2. Physical patient attributes were used as indicators of fluid balance status in nine articles [17–25] including dry mouth, lips, tongue, eyes and/or change in skin turgor. Vivanti [17] reported that amongst 130 clinical variables, tongue dryness was most strongly associated with poor hydration status with a sensitivity of 64%, (95% CI 54–74%) and specificity of 62%, (95% CI 52–72%); however this was used as an indicator of dehydration rather than as an assessment of risk of poor oral fluid intake in patients who did not yet require fluid supplementation.

**Table 2:** Main clinical associations with dehydration from all articles

Confusion or change in mental state [19–26, 28–30]
Diarrhoea and/or vomiting [19–21, 23, 24]
Diuretics [18–21, 23, 26, 28]
Dry mucosa and/or change in skin tugor [17–25]
Fever [18, 20, 21, 23, 24]
Hypotension [18–21, 23, 24, 31]
Physical barriers to drinking [17–19, 21, 23, 26–28]
Poor fluid intake observed [19–22, 24, 25, 27, 29, 32]
Urine appearance [20, 21, 24, 31, 33]

Oral fluid intake barriers were highlighted in eight articles [17–19, 21, 23, 26–28] including swallowing difficulties, physical assistance needed to drink and frequent spills, there was no consensus regarding a definition or bedside assessment process. The inclusion of recent diarrhoea and/or vomiting within a risk assessment was suggested by five articles [19–21, 23, 24]; however these acute symptoms are likely to prompt intravenous fluid replacement on admission to hospital and may not be helpful as indicators that further support for drinking is required.

Confusion or change in mental state was an indicator of risk in 11 articles [19–26, 28–30]. Mentes and Wang [26] reported that 61/133 dehydrated patients had a Mini Mental State examination (MMSE) score of less than 24/30, of whom 40 had dementia. During an intervention with residents receiving verbal prompts, Simmons [30] identified that those with greater cognitive impairment demonstrated a greater fluid intake response.

Low blood pressure or a weak pulse was highlighted in seven articles [18–21, 23, 24, 31] as a useful indicator of dehydration already being present. Vivanti [18] found that a fall in systolic blood pressure whilst standing was separately associated with hydration status. Although fever was described as an independent factor, there was no agreed definition or separation from possible effects upon blood pressure and mental state [18, 20, 21, 23, 24].

An increased risk associated with diuretics was discussed in seven articles [18–21, 23, 26, 28]. Mentes and Wang [26] found that 51/133 dehydrated patients were taking diuretic agents, the results showed that further scrutiny was needed as a negative association with poor oral fluid intake was found during factor analysis. The authors suggested that diuretics may also stimulate fluid consumption relative to the increased output.

Fluid intake volume was used as a risk indicator by nine articles [19–22, 24, 25, 27, 29, 32]. In the South Essex Partnership University NHS Foundation Trust, Food First tool (“GULP”) [20] an individual’s overall risk score was weighted by their 24 h oral intake: zero points >1600 ml; one point 1200 ml–1600 ml; two points < 1200 ml. In Keller’s [32] audit of care homes the protocol for residential care sites for a patient deemed at risk of dehydration was an intake < 1600 ml per 24 h. Kositzke, Zembrzuski and NHS East of England [21, 22, 24] proposed guidelines that staff should encourage a daily intake of at least 1500 ml or 30 ml/kg for patients aged over 60. Similarly Wotton [19] recommended calculating daily intake requirements at 30 ml/kg whilst taking into account co-morbidities and the on-going response to hydration measures. It was not surprising that urine volume and colour was also reported as an important association with dehydration [20, 21, 24, 31, 33], there was no agreement about the length of time for observation or thresholds for changing the fluid support strategy.

### Identification checklist/chart

A formal checklist for dehydration risk was described by ten articles. Eight are summarised in Table 3. Keller [32] has not been included as individual data items were not listed and Bulgarelli [34] used the Mentes and Wang [26] checklist, which is described.

**Table 3:** Checklist for dehydration risk

	Vivanti 2010 [17]	Vivanti 2008 [18]	Wotton [19]	Mentes and Wang [26]	Zembrzuski [21]	NHS East of England [22]	GULP [20]	Mentes and Iowa-Veterans [23]
History								
Age	X	X	✓70+	✓85+	✓85+	X	X	✓85+
Constipation/incontinence	X	X	X	✓	✓	X	X	✓
Diarrhoea/vomiting	X	✓	✓	X	✓	X	✓	✓
Dietary restrictions	X	X	X	X	✓	✓	X	✓
Difficulty swallowing	X	X	✓	✓	✓	X	X	✓
Dizziness/light-headedness	X	X	X	X	X	X	✓	X
Feeling thirsty	✓	✓	✓	✓	✓	✓	X	✓
Low mood	X	X	X	✓	X	X	X	✓
Medication	X	✓	✓	✓	✓	✓	✓	✓
Multiple medical conditions	X	X	✓3+	X	X	X	X	✓4+
Poor mobility/ falls/weakness	✓	✓	✓	✓	✓	X	✓	✓
Pain	✓	X	X	X	X	X	X	X
Recent hospitalisation	X	X	✓	X	✓	X	X	X
Repeated UTIs/Infections	X	X	✓	✓	✓	X	✓	✓
Visual difficulties	X	X	✓	X	X	X	X	X
Observation								
24 hr fluid intake/output	X	✓	✓	X	✓	✓	✓	✓
Blood pressure/pulse	✓	✓	✓	X	✓	✓	✓	✓
Confusion	X	✓	✓	✓	✓	✓	✓	✓
Drowsy/lethargic	X	X	X	X	✓	X	✓	X
Dry mouth/tongue/eyes/skin	✓	✓	✓	X	✓	✓	✓	✓
Fever	X	✓	✓	X	✓	X	✓	✓
Increased respiration	X	✓	✓	X	X	X	X	X
Low body weight/Malnutrition	✓	✓	✓	✓	✓	✓	X	✓
Open wound	X	X	X	X	X	✓	✓	X
Sweating	X	X	✓	X	✓	✓	X	✓
Bedside test								
Hyperglycaemia	X	X	✓	X	X	X	✓	X
Urine								
Colour	X	✓	✓	X	✓	✓	✓	✓
Gravity	X	X	✓	X	✓	X	X	✓
Score Performance								
Sens %	Unknown	Unknown	Unknown	Internal Consistency Theta coefficient 0.68	Unknown	Unknown	Unknown	Unknown
Spec %	Unknown	Unknown	Unknown	Unknown	Unknown	Unknown	Unknown	Unknown
Inter-rater reliability %		83–87%	Unknown	KMO 0.6	Unknown	Unknown	Unknown	Unknown
Compliance %		Unknown	Unknown	Unknown	Unknown	Unknown	Unknown	Unknown
Dehydration present			N/A	Factor loadings for the questions ranged from −0.012–0.567	N/A	N/A	N/A	N/A
Hydration response protocol	X	X	✓	X	✓	✓	✓	✓

Table 3 describes the checklists according to three component categories: history, observation and bedside test. There was a large variation in the size and complexity. In patient history, feeling thirsty, medications and poor mobility/falls/weakness were included in a combination of seven of eight assessments for each factor, whilst diarrhoea/vomiting and repeated UTI’s/infections were included in a combination of five of eight assessments. In observation, blood pressure/pulse, confusion, dry mouth/tongue/eyes/skin and low body weight/malnutrition were included in a combination of seven of eight assessments, whilst 24 h fluid intake/output was included in a combination of six and fever included in a combination of five assessments. Six of the eight assessments included investigating urine colour as a bedside test in the assessment of dehydration risk.

Of the ten articles, five [17–19, 26, 34], did not suggest a clinical response protocol or recommendations for patients at risk. Although Wotten [19] conducted a review of literature and created a risk assessment, there was no clear method described for the selection of included literature and no evaluation.

Mentes and Wang [26] conducted a retrospective analysis to make adjustments to an existing Dehydration Risk Appraisal Checklist (DRAC) containing 40 items including age, health conditions, medications, laboratory results and intake behaviours. This was reduced to 17 questions by conducting an analysis on two previous studies of 133 participants. Overall there was low to moderate association with dehydration. The authors concluded that the analysis supported clinical use of the DRAC whilst highlighting the restricted interpretation due to the small sample size and the additional importance of applying contextual information. Bulgarelli [34] also evaluated the DRAC, a small sample of 21 patients were scored using the checklist within 3 days of admission. Scores on the DRAC did not significantly change between admission and discharge.

Vivanti [18] looked at over 40 clinical, haematological and urinary biochemical parameters employed by medical officers during dehydration assessment in hospital. There were no serial measurements. The parameters were identified through literature; interviews and focus groups. The dominant factor was tongue dryness (OR 4.42; 95% CI 0.86 to 26.10), which would mainly indicate a need for current additional fluid replacement rather than a future risk of poor intake, although it would be expected that there is an overlap between these patient groups.

### Identification checklist/chart with care intervention

An identification checklist with a specific or general care intervention was described by the remaining five articles [20–23, 32]. The GULP tool [20] recorded a score from 0 to 7 points for three categories (24 h fluid intake; urine colour; clinical risk factors for dehydration) and directed the user to present the patient with a matching hydration management plan. The plan included providing information leaflets, engaging the patient in self-monitoring of urine and verbal prompts. The plan development was not reported and there were no data describing its use.

NHS East of England [22] developed a fluid care bundle including an audit tool, patient information and nine principles to assist with fluid management: focus on individual patient needs; assess all patients; facilitate hydration; maintain accurate fluid balance; provide guidance documents for staff; provide information leaflets for patients and relatives; communicate relevant changes in the patient condition; perform fluid assessment audit; analyse fluid related adverse events. No data were presented regarding the bundle impact upon practice.

Zembrzuski and Mentes [21, 23] both summarised published literature to recommend development of local checklists, implementation approaches and individual management plans which included a statement regarding the frequency that patients should be offered drinks. The method of literature selection was not reported and management plans were not tested in clinical practice.

Keller [32] conducted an audit in nursing homes to assess the implementation of a hydration management protocol introduced in three phases: 1) document a dehydration risk, 2) monitor fluid intake for those at risk and 3) aim for >1600 ml intake per 24 hr period. In the first phase 96 records were audited. Due to funding restrictions only 15 records were subsequently examined. Results showed an improvement in compliance for risk documentation (40 to 100%) but no patients achieved the standard set for phases two and three.

### Identification by urinary inspection

Identification of dehydration by urine characteristics was described by two articles [33, 35]. Mentes [33] found significant correlations between researcher ratings on a urine colour (Ucol) chart and urine specific gravity (Usg) for 98 nursing home residents. They proposed that Ucol alone could only be used to cautiously assess hydration status in older adults with adequate renal function (Cockcroft-Gault estimated creatinine clearance [CrCl] values of > or =50 ml/min) as the inter-rater reliability was average to good.

Rowat [35] conducted a small observational study to assess if bedside Usg and Ucol charts were useful indicators of dehydration following acute stroke. Results were compared to urine refractometer readings and routine blood urea:creatinine ratios for 20 patients over a 10 day period. Nine patients developed clinical symptoms of dehydration according to nurse opinion, and although there was good agreement with urine refractometer readings, authors concluded that bedside urine inspection did not provide an early warning of dehydration according to routine U:C ratio measurements.

A further six articles included measurement of Usg or Ucol as indicators of dehydration within their recommendations or tools, no new data were presented [20–22, 24, 31, 33].

### Promotion of oral intake

Wakeling [27] introduced a “hands free” hydration plan for 313 patients in hospital: a bottle was clipped onto the bed with a flexible bite valve hose or patients with greater independence were provided with a plastic sports bottle. In a before and after study using a convenience sample of 313 patients (171 before and 142 during implementation) there was a reduction in length of stay (41 vs. 33 days), dehydration (31 vs. 28 patients) and infections (1 vs. 0 patients). No statistical analysis was performed. The documentation of fluid intake also improved, creating uncertainty about the mechanism of action of the un-blinded intervention.

In nursing homes, regular prompts to drink by a healthcare attendant with or without a beverage cart reduced the frequency of dehydration observed by three studies [29, 30, 36]. Robinson also found a reduction in falls, UTI’s and the use of laxatives. Simmons reported that 81% of participants showed small increases in their average daily fluid intake in response to additional verbal prompts, particularly residents with greater cognitive impairment. 21% also required preference elicitation to increase their intake, mainly amongst residents with less cognitive impairment.

### Professional knowledge/awareness improvement

The relevance of professional knowledge/awareness was described by seven articles [24, 25, 28, 31, 37–39]. Beattie [39] reported a mean score of 4.7/10 from a cross sectional survey of 76 employees to assess knowledge regarding the nutritional needs of nursing home residents. Higher scores were obtained for questions relating to risk factors associated with malnutrition, less than half of respondents regularly recorded fluid intake and only 15% exhibited correct knowledge of fluid requirements.

The English National Health Service (NHS) Nutrition Now Campaign, was promoted by the National Patient Safety Agency (NPSA) and Royal College of Nursing (RCN) comprising 20 points to encourage hydration, fact sheets, care pathway checklists and general advice. There was no supporting information regarding the development of the fact sheets or their impact [38].

Survey results from 53 lead nurses (a 33% response rate) undertaken by NHS Kidney Care regarding the use of a poster campaign to promote hydration, showed that although 70% of respondents had displayed the posters, only 45% had a policy to monitor hydration, 15% felt their local policy needed updating, and 11% did not have a policy. Respondents identified hydration monitoring challenges including compliance with documentation, keeping practice up to date and staff awareness [37].

## Discussion

Prevention of dehydration amongst vulnerable populations remains a healthcare priority. The National Institute for Healthcare and Clinical Excellence [7] proposed that 12,000 cases of acute kidney injury could be avoided with pro-active fluid management. Pash [6] found significant differences in costs and length of stay associated with dehydration in hospital ($33,945 vs. $22,380 and 12.9 vs. 8.2 days). Nursing assessments are routinely used to document a risk of pressure ulcers and malnutrition, so it is surprising that there is no standardised assessment to identify older persons at risk of inadequate fluid intake following a change in health status or care setting.

The results of our review confirm that dehydration prevention activities are not informed by strong evidence, and most studies have focused upon identification of patients who are already in negative fluid balance. Some authors described statistical isolation of characteristics associated with dehydration. Their conclusions were limited due to the small sample size, unclear environmental context, and lack of an accompanying response protocol to demonstrate clinical value. They reported challenges when balancing the practicality of an effective, single bedside, dehydration risk assessment against the number of factors which may be relevant for different patient groups, across different settings. Therefore it is currently not possible to recommend a specific assessment. Previous reviews [2, 11] found that there was no ideal single combination of risk factors and to avoid dehydration recommended the use of routine fluid balance monitoring combined with, improvements in beverage choice, staff awareness and assistance with toileting (to prevent the avoidance of drinking). The reliability and impact for resources of performing long term routine fluid balance monitoring on all patients has not been evaluated and may not be necessary if there is better recognition and targeting of vulnerable groups.

We did not include in our review, studies which were evaluating new technology to assess current fluid status, as our focus was prediction of poor fluid intake using clinical information at the bedside. The recent Cochrane [11] review has suggested that further study in this area may be useful, for example, BIA at resistance of 50 kHz of total body water. In terms of screening for impending water loss dehydration the Cochrane review found that potentially useful tests were missing some drinks between meals and expressing fatigue, whereas it was not useful to observe urinary measures, orthostatic hypotension, skin turgor, capillary refill, dry mouth assessments, sunken eyes, thirst and headache. It has recommended that some of this information could be combined to contribute towards a useful predictive instrument, but further research is required. During routine care at the bedside, pulse volume and blood pressure readings can provide an opportunity to identify some patients with dehydration; these also reflect current health state and may not separately indicate a risk of poor oral intake. An intake record over a 24 h period was also recommended as helpful for recognising patients at risk, but passive observation alone could lead to delayed intervention and increased use of intravenous fluids. Even after staff training, fluid balance recording can be incomplete particularly for patients with cognitive impairment [27]. Although a statistical association in a single setting has been demonstrated between dry mucosal membranes and objective measures of fluid status, this alone would not necessarily avoid the use of interventions such as intravenous fluid replacement. Examination of urine characteristics as a bedside assessment does not appear to be of additional value.

The single most common risk factor reported with some evidence for a matching behavioural intervention was change in mental state. Nearly half of the patients in the population studied by Mentes [26] scored less than 24 on the MMSE, and in development of a risk assessment Wotton [19] highlighted the importance of papers describing a link between dehydration and poor cognitive abilities. Simmons [30] found that patients with cognitive impairment consumed more fluids after an increase in verbal prompts, whilst Robinson [29] reported that using brightly coloured cups and beverages, with different tastes and temperatures was well received.

The care interventions identified appear to indicate that the provision of extra opportunities such as a beverage cart to prompt and/or receive drinks is a modifiable factor in the maintenance of hydration. These simple interventions would be easy to implement and lend themselves to further research, ideally with a cluster trial design to control for clinical service and population variations. With the introduction of nutritional assistants onto some NHS hospital wards, the wider short and long term impact on dehydration prevention could be investigated [40].

Although there is evidence that healthcare staff knowledge about fluid requirements and hospital policies could be improved, behavioural approaches driven by individual patient assessment and local audit, are more likely, to be more effective in changing care delivery than simply providing more information to staff or short term national campaigns [41].

The mixture of settings, terminology and observation/ intervention approaches used by articles identified from the search strategy, provided a challenge when summarising the available evidence and guidance, and we have attempted to give the results clinical relevance. Due to the specific focus upon fluid intake, we cannot be sure that relevant information was not included from research with a more nutritional focus. We concentrated upon institutional settings as this would have the greatest relevance for patients at highest risk of dehydration, but it is possible that there may also be literature relating to maintaining hydration in the community.

## Conclusion

The clinical assessment of dehydration status and risk has been promoted by researchers, policy makers and health improvement agencies but without a co-ordinated or evidence-based approach. Individuals with cognitive impairment are at greater risk and may respond to increased opportunities and support for drinking. Urine inspection does not appear to be of routine value. Simple care interventions to encourage oral fluid intake can be effective, to save resources these should be targeted at highest risk groups identified, particularly individuals with cognitive impairment. There is a need to emphasize the importance of hydration, making it a collective responsibility through staff education, clinical documentation, and hospital policy and audit systems.

## Additional file

Additional file 1: Search strategy – Provides the search strategy followed for MEDLINE, EMBASE and CINAHL databases (DOCX 16 kb)

## Abbreviations

BIA: Bioelectrical impedance analysis
DRAC: Dehydration risk appraisal checklist
MMSE: Mini mental state examination
NHS: National health service
NPSA: National patient safety agency
RCN: Royal College of nursing
Ucol: Urine colour
Usg: Urine specific gravity
