# Supplementary material for: Clinical assessments and care interventions to promote oral hydration amongst older patients: a narrative systematic review
Source: BMC Nurs. 2017 Jan 17;16:4. doi: 10.1186/s12912-016-0195-x (PMC5240391; doi:10.1186/s12912-016-0195-x)
Supplement: Additional file 1: — Search strategy – Provides the search strategy followed for MEDLINE, EMBASE and CINAHL databases (DOCX 16 kb) [file 12912_2016_195_MOESM1_ESM.docx]

**Additional file 1 Search Strategy**

| No. | MEDLINE/EMBASE | CINAHL |
| --- | --- | --- |
| 1 | Dehydration.mp. [mp=ti, ab, tx, ct, hw, sh, tn, ot, dm, mf, dv, kw, nm, kf, px, rx, ui] | Dehydration |
| 2 | Hydration.mp. [mp=ti, ab, tx, ct, hw, sh, tn, ot, dm, mf, dv, kw, nm, kf, px, rx, ui] | Hydration |
| 3 | Drinking.mp. [mp=ti, ab, tx, ct, hw, sh, tn, ot, dm, mf, dv, kw, nm, kf, px, rx, ui] | Drinking |
| 4 | Hospital$.mp. [mp=ti, ab, tx, ct, hw, sh, tn, ot, dm, mf, dv, kw, nm, kf, px, rx, ui] | Hospital$ |
| 5 | Care home$.mp. [mp=ti, ab, tx, ct, hw, sh, tn, ot, dm, mf, dv, kw, nm, kf, px, rx, ui] | Care Home$ |
| 6 | Nursing home$.mp. [mp=ti, ab, tx, ct, hw, sh, tn, ot, dm, mf, dv, kw, nm, kf, px, rx, ui] | Nursing Home$ |
| 7 | Assessment.mp. [mp=ti, ab, tx, ct, hw, sh, tn, ot, dm, mf, dv, kw, nm, kf, px, rx, ui] | Assessment |
| 8 | Tool.mp. [mp=ti, ab, tx, ct, hw, sh, tn, ot, dm, mf, dv, kw, nm, kf, px, rx, ui] | Tool |
| 9 | Screening.mp. [mp=ti, ab, tx, ct, hw, sh, tn, ot, dm, mf, dv, kw, nm, kf, px, rx, ui] | Screening |
| 10 | Evaluation.mp. [mp=ti, ab, tx, ct, hw, sh, tn, ot, dm, mf, dv, kw, nm, kf, px, rx, ui] | Evaluation |
| 11 | Rating scale.mp. [mp=ti, ab, tx, ct, hw, sh, tn, ot, dm, mf, dv, kw, nm, kf, px, rx, ui] | Rating Scale |
| 12 | Older people.mp. [mp=ti, ab, tx, ct, hw, sh, tn, ot, dm, mf, dv, kw, nm, kf, px, rx, ui] | Older People |
| 13 | Elderly.mp. [mp=ti, ab, tx, ct, hw, sh, tn, ot, dm, mf, dv, kw, nm, kf, px, rx, ui] | Elderly |
| 14 | Prevention.mp. [mp=ti, ab, tx, ct, hw, sh, tn, ot, dm, mf, dv, kw, nm, kf, px, rx, ui] | Prevention |
| 15 | 4 or 5 or 6 | S4 or S5 OR S6 |
| 16 | 12 or 13 | S12 or S13 |
| 17 | 1 or 2 | S1 or S2 |
| 18 | 7 or 8 or 9 or 10 or 11 | S7 or S8 or S9 or S10 or S11 |
| 19 | 3 or 17 | S3 or S17 |
| 20 | 14 and 15 and 16 and 18 and 19 | S14 and S15 and S16 and S18 and S19 |
| 21 | Remove duplicates from 20 |  |
